# A Systematic Review on the Prevalence and Risk of Orthorexia Nervosa in Health Workers and Students

**DOI:** 10.3390/ijerph21081103

**Published:** 2024-08-21

**Authors:** Ellie G. McInerney, Peta Stapleton, Oliver Baumann

**Affiliations:** School of Psychology, Bond University, Robina, QLD 4226, Australia; ellie.mcinerney@student.bond.edu.au (E.G.M.); pstaplet@bond.edu.au (P.S.)

**Keywords:** orthorexia, health career, health fields

## Abstract

Extensive knowledge on nutrition and dieting has been associated with an increased risk of orthorexia (an obsession with food that one considers healthy) within the research literature. A systematic review was conducted to identify the prevalence of orthorexia in individuals who were employed in, or studying, health careers. The databases Psychinfo and Pubmed were searched to obtain research articles. Studies assessing orthorexia and either students or workers within health-related fields were included. A study merit rating system was utilised to assess the quality of each study included. In total, 26 articles were included in the current review after applying inclusion and exclusion criteria. The prevalence of orthorexia was the greatest in dietetic students. Research findings consistently demonstrated that the prevalence of orthorexia was higher in those working or studying in health fields than the general population. Individuals in their early stages of study or career appear most at risk. Orthorexia prevalence, however, does not appear to differ by sex or BMI. Orthorexia is novel research area. The trends in the current review suggest that individuals in health-related careers appear to be more at risk of orthorexic patterns. More research in this area is needed through the use of scales with greater psychometric properties.

## 1. Introduction

A systematic review conducted by Galmiche et al. [1] revealed that the lifetime prevalence of eating disorders is 8.4% for women and 2.2% for men. Galmiche et al. [1] also identified that eating disorder prevalence has been gradually increasing over time. Within the Australian population, it is estimated that 16.3% of individuals suffer from eating disorders or disordered eating [2]. Eating disorders have a particularly high prevalence within the university population (51.8% in women and 31.3% in men) [3]. It has been reported that the incidence of various eating disorders increased during the COVID-19 pandemic, increasing by 67% in males and 83% in females [4,5]. Despite this high prevalence, it is likely these results underestimate the true prevalence statistics due to underdiagnosis and underreporting [6].

Currently, in the Diagnostic and Statistical Manual of Mental Disorders—Fifth Edition (DSM-5; [7]), there are several formally recognised feeding and eating disorders: pica, rumination disorder, avoidant/restrictive food intake disorder, anorexia nervosa, bulimia nervosa, binge-eating disorder, and eating disorders ‘not otherwise specified’. Beyond these recognised conditions, the term disordered eating has been used to acknowledge individuals’ maladaptive and atypical eating patterns which lie outside the specific criteria of a feeding and eating disorder.

Research has previously identified that students within the health science field demonstrate an increased prevalence of disordered eating. In particular, nutrition and dietetics students appear to exhibit the greatest prevalence of disordered eating behaviours [8,9].

It has been hypothesised that nutrition students may demonstrate greater disordered eating patterns than their peers, which are evident even prior to the commencement of studies [10]. This background of food fixation may then act as a motivating factor to commence nutritional studies. Whilst undertaking a nutrition degree, an individual’s knowledge of nutrition is further enhanced, which may therefore perpetuate and intensify pre-existing predispositions for food obsessions and fixations [10].

Research has identified a positive correlation between an individual’s eating habits and their level of nutritional knowledge [11,12], such that individuals who currently or have previously received education on nutrition and dietetics are at a higher risk of engaging in obsessive behaviours regarding healthy eating. These maladaptive obsessions lie within a scope wider than the currently acknowledged eating disorders [11]. The obsessions demonstrated by these populations do not always surround food quantity but instead are often focused on food quality. It therefore appears that the current diagnostic domains for eating disorders may be only partially capturing the true topography of maladaptive eating behaviours, particularly within this population. The maladaptive behaviours described in the referenced literature are more congruent to the novel orthorexic disorder.

The term orthorexia was first coined by Bratman in 1997 [13]. He used the term to describe an abnormal pattern of behaviour where individuals experience a maladaptive obsession with healthy eating [13]. According to the current literature, individuals suffering with orthorexia severely restrict their diet, and typically will only consume pure or whole foods which are not genetically modified, free of grains, low in salt or sugar, free of pesticides, organic, and low in fat [14,15]. These individuals are also reported to generate self-imposed dietary rules, according to proposed diagnostic criteria by Dunn and Bratman [16].

In Bratman’s [17] more recent work, he proposes that those with orthorexia tend to demonstrate an increasingly narrow range of food that they consider ‘healthy’ and show a preoccupation with the same foods, leading to interferences in several aspects of their life, causing social and occupational impairments. Furthermore, Bratman [17] cites that when there is a violation of these self-imposed food rules, individuals suffering with orthorexia experience significant anxiety and guilt. Such outcomes suggest the presence of rigid maladaptive thinking styles congruent with presentations of other eating disorders, namely anorexia.

Despite the maladaptive cognitions, attitudes, and behaviours of orthorexia and the consequential impacts on social, occupational, and psychological functioning, orthorexia is not currently acknowledged as a formal diagnosis in the DSM-5 (i.e., only listed in the Appendix). Due to the novelty of the disorder, a large degree of ambiguity persists surrounding the symptoms, prevalence, and risk factors of the disorder.

Several tools have been developed to assess orthorexia, each with its own strengths and limitations. The ORTO-10, ORTO-11, and ORTO-15 scales, developed by Donini and colleagues, are among the most commonly used tools and measure orthorexic tendencies through self-reported behaviours and attitudes towards food. The ORTO-15 is particularly widespread in research; however, it has faced criticism for low internal consistency, inconsistent cut-off points, and questionable construct validity. The ORTO-11 and ORTO-13, which are shorter versions, share similar psychometric challenges. The Düsseldorf Orthorexia Scale (DOS), developed by Barthels and Pietrowsky [18], focuses on the pathological aspects of orthorexia and has shown good psychometric properties, but its generalizability across cultures is still under evaluation. The Eating Habits Questionnaire (EHQ) by Gleaves et al. [19] offers a broader perspective by assessing emotional and social aspects of orthorexia, but it may lack comprehensive validation across different populations. The Teruel Orthorexia Scale (TOS), developed by Barrada and Roncero [20], differentiates between healthy orthorexia and orthorexia nervosa, but it too requires further validation in diverse populations. A systematic review by Opitz et al. [21] highlighted the variability in the psychometric properties of these tools, indicating the need for caution when interpreting results based on these measures and emphasising the importance of developing more reliable assessment tools for future research.

Considering the current literature, which suggests that a preoccupation in healthy eating and health culture is a risk factor for the development of orthorexia, this systematic review aimed to evaluate the current literature in the field and draw conclusions regarding the prevalence of orthorexia in individuals who study or work in health-related careers. By researching and evaluating the currently available literature, this systematic review sought to analyse the current quality and thus reliability of findings in this field. This review also aimed to explore whether individuals who work or study in health-related fields are more likely than those in non-health fields to develop orthorexia. It was hypothesised that due to an increased interest and learnt knowledge regarding health and diet individuals with health careers experience, those in health-related studies and occupations would demonstrate more orthorexic tendencies and symptoms, as in line with the research conducted by Korinth et al. [10].

This systematic review utilised psychological databases to identify studies that assessed the prevalence of orthorexia in health-related studies and occupations. Relevant publications were then accumulated and rated based upon research quality. The findings and implications of the accumulated studies were synthesised and grouped, allowing for the identification of general trends and patterns within the literature field.

## 2. Materials and Methods

### 2.1. Literature Search

The literature search included papers written in English which explored the prevalence of orthorexia in individuals studying or working within the health field. The electronic databases PsycINFO and PubMed were systematically searched from database inception until 12th of May 2022. The following keywords were used to search the databases orthorexia, students, dietitians, nutritionist, doctor*, physio*, nursing, and psycholog*. ‘Orthorexia’ was combined with each health field term by using ‘AND’ in each database. Additional papers were also obtained from the reference lists of publications identified in the initial search.

### 2.2. Selection Criteria

#### 2.2.1. Inclusion Criteria

As the purpose of the current review is to analyse the prevalence and risk of orthorexia in health fields, all publications were required to assess orthorexia as a dependent variable. Orthorexia was defined by meeting the following criteria: it involved obsessive behaviours, focused on food quality, and included a statement indicating impaired functioning. The populations of both students and workers within health-related fields were included in the review. The publications were required to extrapolate between the health population and other students/workers. All publications were required to have obtained empirical data. Studies were required to specify the name of each utilised measure. Only studies which were published in peer-reviewed journal articles were included. All studies were required to be written in the English language.

#### 2.2.2. Exclusion Criteria

Any publication which did not analyse orthorexia as a dependent variable, such as studies which aimed to validate orthorexia scales, were excluded from the current review. Review papers were excluded from the current review.

### 2.3. Data Extraction

In order to ensure a systematic approach to the research and appraisal of the relevant publications, all data were extracted in a consistent process. This extraction process ensured that all inclusion and exclusion criteria were met. From all applicable results, the following extractions were made: the sample demographics; type of sample; study design; instruments utilised; statistical analyses used on data; findings, including the direction and magnitude of effect sizes; and limitations.

In addition to this, a scientific merit rating scale developed by Waxman [22] was utilised to assess the quality of each included publication. The Waxman [22] quality rating scale evaluates the methodological quality of studies using the following six criteria:

1. Presence of a Control Group: This criterion checks whether the study includes a control group for comparison with the experimental group.
2. Matching of the Control Group: This evaluates whether the control group is appropriately matched to the experimental group on key variables such as age, gender, and other relevant factors.
3. Representativeness of the Sample: This assesses how well the study sample represents the population from which it was drawn, considering factors like sample size and selection method.
4. Definition of the Eating Disorder (ED) Sample: This criterion checks whether the ED sample is clearly defined, including diagnostic criteria and any subtypes within the sample.
5. Reliability of Instruments: This evaluates whether the tools and measures used in the study are reliable, meaning they consistently produce similar results under the same conditions.
6. Appropriateness of Limitations: This checks whether the study appropriately acknowledges and discusses its limitations, providing context for the findings.

Each of these dimensions is scored as either 1 (present) or 0 (absent), and the total score is summed to create a quality rating with a possible range of 0 to 6.

The rating outcomes can be identified in the results section of the review.

For inter-rater reliability purposes, both authors separately conducted the literature search and publication quality ratings. Independent results were compared and revealed nil inter-rater discrepancies.

## 3. Results

### 3.1. Surface Characteristics

**Figure 1:**
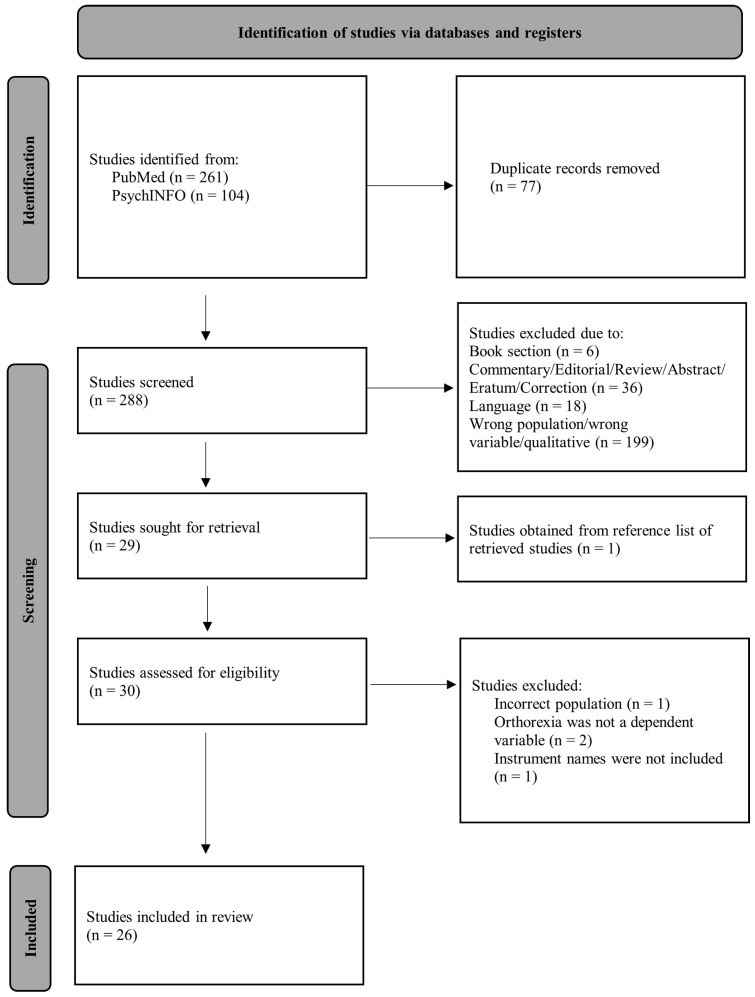
Prisma chart of study selection process.

Following a comprehensive search and selection process, 26 studies were deemed applicable for the current review. A visual depiction of the literature search and process of obtaining suitable articles can be viewed in the Preferred Reporting Items for Systematic Reviews and Meta-Analyses (PRISMA) chart in Figure 1. All publications were found based upon the aforementioned search criteria, with the exception of one study, which was found in the reference list of one of the other publications included in this review.

Of the included papers, one study was Greek, three studies were from Poland, eleven from Turkey, one was from Sweden, one was Austrian, one from Jordan, two were German, two Italian, one from Lebanon, one from the United States of America, one was Chilean, and one was Brazilian. The publication dates ranged from 2006 through to 2022. All studies utilised a cross-sectional design.

Table 1 provides a summary of included study characteristics. Information, such as the author, date, sample characteristics, assessment tools, main results, and limitations, is included.

### 3.2. Quality Assessment

Each of the studies included in the current review were assessed for quality and provided a quality rating score. This method was adopted by Waxman [22] which is based upon methodological criteria initially developed by Eiser et al. [47]. The quality ratings can be viewed in Table 2. The quality rating scores ranged from one to five. There was a mean quality rating score of *M* = 2.65. This mean score indicates that overall, the quality of the literature in this field is within the average range. Such finding is somewhat expected given the infancy of this research area.

Each of the rated areas were operationalised to provide clear rating criterion. A sample was determined representative if it employed stratified sampling techniques as opposed to convenience sampling methods. Due to the fact that there are currently no diagnostic criteria for orthorexia, orthorexia was determined well-defined if it met the following parameters: there was reference to obsessive behaviours, reference was made to food quality, and there was a statement regarding associated impaired functioning. The mention of functional impairment is vital, as such outcomes mediate the difference between typical and pathological behaviours [48]. These parameters were chosen as they are the most widely agreed upon diagnostic components within the literature.

To be rated as having utilised sound psychometric assessments, the study variables most salient to the research were required to be measured by psychometrically sound tools. Due to the extensive use of the ORTO-11 and ORTO-15 psychometric tools, which have varying evidence attesting to their psychometric properties, research papers which used these assessments were required to also employ a secondary measure of orthorexia that had to be rated as employing sound psychometric assessments [49]. It is important to recognise that the inclusion of a secondary measure does not guarantee that orthorexia was rated according to gold standard diagnostic criteria (see Alshaibani et al.) [50]. Nonetheless, this approach acknowledges that the authors of these studies are aware of the shortcomings of the ORTO scales and have attempted to mitigate these by including a secondary measure. For a further discussion of the limitations of the current diagnostic approaches for orthorexia, please see the discussion section.

For the final rating criteria, the most notable limitations, which were at the greatest risk of undermining study findings, were required to have been addressed by the publication.

## 4. Discussion

### 4.1. Prevalence Themes

After analysing the reviewed studies, the following prevalence themes were recurrently identified: sex differences, body mass index (BMI) determinants, global prevalence of orthorexia, and career specific prevalence rates. In this section, the initial exploration is centred around participant attribute variables and their relationships to orthorexia; following this, consideration is given to orthorexia prevalence within the specific health populations.

### 4.2. Sex Differences

The majority of the reviewed literature supports the notion that there are no sex differences in the prevalence of orthorexia within health-related professions [8,10,30,36,40,41]. Contrasting with this finding, some limited literature exists which supports a significant differentiation in orthorexia prevalence between the sexes. Abdullah et al. [23] found that amongst nutritionists and nutrition students, prevalence rates were significantly greater in males. Conversely, Sanlier et al. [42] found that in their study of university students, females had a significantly greater risk of orthorexia, which was cohobated by later research by Aktürk et al. [25]. However, due to the limited number of studies which were able to identify significant sex differences, the findings are not currently robust enough to suggest true sex differences. The current body of literature indicates that there are nil sex biases in orthorexia prevalence for those within health-related fields of study or occupation.

### 4.3. BMI

The evidence regarding associations between BMI and orthorexia lacks uniformity. Some research has found significant positive relationships between BMI status and orthorexia [8,23], whilst a larger volume of evidence indicates a negative relationship between the two [26,29,34,44,45]. Despite some evidence of association, the majority of research articles have found nil indication of any significant relationships between BMI and orthorexia [10,36,37,40,41,42,46].

Dittfeld et al. [31] conducted a study examining orthorexia in dietetic and physiotherapy students. They found that there was no significant relationship between BMI and orthorexia for physiotherapy students, whilst the dietetic students demonstrated modest significance, with higher BMIs being associated with a higher degree of orthorexia prevalence. A study by Surała et al. [51] utilised a participant group of competitive athletes and found that for males, the orthorexia scores were positively related to BMI but only for athletes who competed in weight-dependent sports. Such findings indicate that although associations between BMI and orthorexia can be achieved when the sample groups are further refined, these findings are not marked or robust. Currently, there is nil indication to support any prominent relationship between BMI and orthorexia. When reviewing the literature at large, any effects identified in research appear to be small and innocuous.

### 4.4. Global Prevalence

Throughout the collated research, the global prevalence of orthorexia within health-related careers and areas of study appears to vary greatly. Across the reviewed literature, the lowest prevalence rate was 3.3% with the greatest prevalence rate being 80% [30,35]. The majority of the prevalence statistics fell within a range of 28–76%. Such notable variations in prevalence statistics can in part be attributed to the varying instruments and cut-off points utilised in the studies. These differing instruments also fluctuate in their psychometric soundness, which must be taken into consideration.

These notable prevalence outliers can be explained through the analysis of methodology techniques. Depa et al. [30], who identified a prevalence of 3.3%, were the only scholars to utilise the Duesseldorf Orthorexia Scale. This scale has demonstrated good psychometric properties with sound internal consistency, test re-test reliability, and construct validity [30]. Prevalence rates using this scale are substantially and continuously lower compared to studies using other orthorexic instruments [18]. Another potential reasoning for this lowered prevalence rating may be due to cultural considerations. The study by Depa et al. [30] was conducted in German with the Duesseldorf Orthorexia Scale being an exclusively German measure. However, even when the measure has been converted into English and utilised in the United States, prevalence statistics are still much greater than when the measure is utilised in Germany [52]. Such findings indicate that cultural aspects may play a role in the differing prevalence statistics, with German populations demonstrating consistently lower rates of orthorexia.

Research by Freire et al. [35], who reported an 80% prevalence rate, utilised a sample of physical practitioners. However, these participants were sampled from gym locations across Brazil. Thus, the sampling environment was an inherent confounding factor which would likely explain, above and beyond their career, the high prevalence of orthorexia. It is likely that those practitioners who have an increased interest and concern with healthy eating would also be more likely to attend the gym. This, therefore, does not provide an adequate generalisation of the prevalence of orthorexia in physical practitioners. Like the aforementioned study, research by Freire et al. [35] also received a rating of two on the study merit rating system; thus, it is important that caution is taken when interpreting these findings.

### 4.5. Career Specific Prevalence

Within the collated research articles, there were three distinct categories of health careers and study evaluated: the area of nutrition and dietetics, nursing and medicine, and general health-related university degrees, which were not specified in the publications.

#### 4.5.1. Nutrition and Dietetics

Fifteen of the reviewed articles utilised populations from the areas of nutrition and dietetics. Within the literature, there was a consistent trend, that although nutrition students demonstrated high prevalence rates of orthorexia, qualified nutritionists demonstrated significantly lower prevalence rates [10,29,44]. Korinth et al. [10] conducted a study which examined first year nutrition students, higher semester nutrition students, and non-nutrition university control students. The first-year nutrition students demonstrated significantly greater dietary restrain than control students. However, orthorexic tendencies significantly decreased in the nutrition students between the first and seventh semester of study, whilst the prevalence remained stable in the control group. This finding was further supported by Asil and Sürücüoğlu [26], who found that the food frequency score indicative of agreement with dietary recommendations did not differ between first year nutrition and control students; however, food selection improved in nutrition students toward the end of their degrees, whilst matched controls demonstrated impaired food selection. Such findings indicate that as students’ progress in their nutrition and dietetic studies and become more educated, their tendency to engage in orthorexic behaviours decrease. This is supported by research that demonstrates low prevalence levels of orthorexia in qualified nutritionists [26,44].

Such findings support the notion that orthorexic tendencies, relevant personality factors such as ridged thinking, obsessive qualities, and interest in nutrition, exist prior to engagement in nutritional studies [53]. These pre-existing maladaptive behaviours may be a motivating factor in them choosing a career in nutrition. The impact of receiving comprehensive knowledge and training on food and nutrition is a secondary factor which may exacerbate pre-existing and pre-pathological orthorexic behaviours. It can be hypothesised that this is especially pertinent during the earlier years of study before individuals acquire critical thinking in the field. Course content in a nutrition degree is often conflicting between subjects [54]. Individuals who already have more rigidity in thinking may have difficulty allowing for these conflicting ideas and multiple truths. Individuals with rigid thinking styles are more likely to take in nutritional information as absolute truths and become fixated on the need to only eat healthily. It appears that students with rigid thinking styles gravitate towards nutritional degrees, with study findings revealing that students who experience obsessions about their body image are significantly more likely to undertake a degree in nutrition [55].

However, the literature trends suggest that after a certain point of study, this knowledge then become protective in minimising orthorexic behaviours and promoting healthier eating habits [45,53]. It could be hypothesised that as individuals progress in their careers and enhance their knowledge, they may employ more critical thinking regarding health information, have greater insight into their own maladaptive eating behaviours, learn to employ more flexible thinking, and those who have highly fixed and ridged thinking styles may not progress to becoming registered dietitians.

Within the research on dietitians, it was found that individuals who had either a past or current eating disorder demonstrated a significantly greater risk of developing orthorexia [28,29]. The prevalence of eating disorders is much greater in dietetic students than other university populations [56]. One research article found that 30% of students who enrolled in a nutrition and dietetics degree had a personal lived experience of an eating disorder [57]. Such findings can aide in the explanation of the high prevalence rates for individuals in nutrition and dietetic programmes who experience orthorexia due to its high comorbidity rates with other established eating disorders.

In line with Bratman’s theory of Orthorexia, Kinzl et al. [38] identified that 8.8% of sampled dietitians gained a sense of increased self-esteem from eating healthy food. Furthermore, 4.6% reported feeling guilty or self-loathing if they did not follow their self-imposed dietary rules. Such results support the notion that individuals who undertake dietetic degrees may have greater ridged thinking styles which are then highlighted throughout their knowledge acquisition and expressed through the development of orthorexia.

#### 4.5.2. Nursing and Medicine

Within the literature on healthcare workers, there were two specific participant groups: nurses and doctors. Only two research papers were published on the relationship between nursing and orthorexia with no identifiable trends [25,43]. Both publications received a one on the merit rating system. Such deficits in research methods may explain the lack of consistent findings. More research in this field is needed before any inferences can be drawn.

A study by Yılmazel [46] found that in their research of both nurses and doctors, doctors had a significantly greater risk of orthorexia than nurses. Furthermore, two research articles corroborated that younger doctors had significantly greater tendencies for orthorexia then older professionals [34,35]. Prevalence within this population was highly varied from 43.6% to 80% [34,35]. Several research articles again identified a significant correlation between orthorexic behaviour and previous eating disorder diagnoses [32,33,34,35].

However, the aforementioned research by Freire et al. [35] had a significant confounding variable, with participants being sampled from gyms across Brazil. Therefore, these results are skewed towards doctors who also work out and are not representative of the medical field at large.

Furthermore, Erol and Özer [32] found that doctors who received information from nutritionists had significantly higher eating disorder rates than those who did not. This supports the hypothesis that nutritional knowledge contributes to orthorexia onset. Bağci Bosi et al. [27] found that 20.1% of males and 38.9% of female medical students stated that their food selection was influenced by nutrition and health advice provided on social media. This finding further supports the notion that an increase in knowledge is associated with an increased risk of orthorexia. It also supports the hypothesis that students are highly influenced by nutritional knowledge and do not engage in critical thinking about the content, as seen with nutrition students.

#### 4.5.3. Health Majors versus Control Students

Participants in health-related degrees were found to have a significantly greater prevalence of orthorexia compared to control students [30,39,40,41]. However, this finding did not hold with research by Sanlier et al. [42] and Guglielmetti et al. [36], who found that there was no difference in orthorexia prevalence between students in health, mathematics, social science, economics, or sports science degrees.

### 4.6. Key Finding

The trends identified in this review suggest that, beyond individuals in health-related careers being more prone to manifest orthorexic patterns of behaviour, there are notable differences when compared to the prevalence of other eating disorders. The prevalence of eating disorders such as anorexia nervosa, bulimia nervosa, and binge-eating disorder varies across different populations. In the general population, the lifetime prevalence of anorexia nervosa is approximately 0.9% for women and 0.3% for men, while bulimia nervosa has a lifetime prevalence of about 1.5% for women and 0.5% for men. Binge-eating disorder is more common, with a lifetime prevalence of around 3.5% for women and 2.0% for men [1].

In health-related occupations, the prevalence of these disorders can be higher due to factors such as occupational stress and body image pressures. For instance, health professionals, particularly those in fields emphasising body image like dietitians and nurses, may have higher rates of anorexia nervosa. Similarly, bulimia nervosa and binge-eating disorder may also be more prevalent among health professionals, influenced by job-related stress and access to food [58].

Unlike these more well-established eating disorders, orthorexia appears to be driven less by body image dissatisfaction and more by an obsession with healthy eating. Interestingly, Plichta et al. [41] found that body satisfaction was not related to the development of orthorexia, contrasting with research findings in bulimia and anorexia. This provides further evidence regarding the differing motivational factors in orthorexia compared to those of established eating disorders. Moreover, a noteworthy correlation between ORTO-15 scores and the usage of the social media platform Instagram suggests that social comparison may contribute to orthorexia via an association with dietary standards rather than body image [59].

### 4.7. Strengths and Limitations

The reviewed studies are valuable due to their novelty in the field, which is relatively under-researched. They have helped to identify at-risk cohorts, preliminary symptoms, and possible diagnostic criteria, contributing to the growth and standardisation of orthorexia research.

While the reviewed publications had limitations, notably their reliance on cross-sectional and correlational designs, preventing causal inferences, they did highlight associations between orthorexia and working in health-related careers. The primary limitation across orthorexia research is the lack of reliable psychometric tools; the widely used ORTO-15 is criticised for not capturing the obsessive aspect of orthorexia, and there is inconsistency in the cut-off points, raising doubts about the accuracy of prevalence assessments [21].

This has led to a substantial number of studies with low merit ratings, limiting their interpretability and validity. Nevertheless, this review offered valuable insights into the emerging field of orthorexia, benefitting from recent research and providing a focused analysis on high-risk populations, which can inform early intervention and awareness efforts. However, it missed examining prevalence in other populations and delving into the psychological variables underpinning orthorexia development, leaving room for future studies to explore these aspects. We recognise the importance of comparing these findings with those from other relevant populations, such as students in non-health disciplines or the general population. The absence of a standard control group is a limitation, and future studies should aim to include more varied comparison groups to better contextualise the prevalence of orthorexia.

Moreover, while there are currently no universally accepted diagnostic criteria for orthorexia, this study has relied on the informal criteria used in existing literature to estimate its prevalence. We recognise this as a limitation and emphasise the importance of future research to refine and standardise diagnostic criteria. Such efforts will be crucial in enhancing the accuracy of prevalence estimates and advancing our understanding of Orthorexia Nervosa as a distinct condition.

Finally, another limitation of this review is the exclusion of studies published after May 2022. While orthorexia research has continued to evolve, our review was designed to provide a thorough analysis of the literature available up to that point. Future research could benefit from incorporating more recent studies to further expand on the findings presented here and to capture the latest developments in the field.

## 5. Conclusions

The current systematic review analysed trends in the literature to identify if orthorexia has a greater prevalence in individuals in health-related studies and occupations compared to those in other career areas. In line with the study hypothesis, it was found that individuals in health-related studies and occupations tend to demonstrate more orthorexic tendencies and symptoms than those in non-health-related fields. Specifically, individuals in their early stages of study or career appeared to be most at risk of developing orthorexia. Namely, nutrition and dietetics students appear to have the greatest prevalence of orthorexia.

The findings of this review have significant implications for both policy and practice. This review highlights the need for students in health degrees to be educated regarding the risks of orthorexia development and available treatment options. Understanding population prevalence is essential for clinical practice to ensure psychologists are cognisant of the potential risks and the populations in which orthorexic symptoms are most likely to occur. It is important to note that, due to the novelty of this research area, there is a substantial need for continued research. Of primary importance is the establishment of diagnostic criteria. Once this is established, research can then focus upon the creation of psychometrically sound assessment tools to assess the presence of orthorexia. This will then provide a more accurate estimate of the current prevalence rates, generally and within specific populations.

A more accurate understanding of population-specific prevalence is crucial for identifying of risk factors and protective factors, which are essential for developing effective treatment guidelines.

## Figures and Tables

**Table 1:** Summary of included study characteristics.

Author and Date	Sample Size and Type of Health Field	Age	Control Group Y/N	Type of Sample	Assessment Tools	Main Results	Limitations
Abdullah et al., 2020 [23]	*N* = 307 undergraduate students of nutrition and dietetics, 82 postgraduate students, 32 graduates and nutritionists Sampled from 6 universities	18 to 22 *M* not stated	N	Stratified	ORTO-15	A total of 72% orthorexia prevalence Mean ORTO-15 = not specified Orthorexia significantly affected by BMI, and gender, with higher male prevalence As BMI increased, orthorexic tendencies increased	Cross-sectional design ORTO-15 psychometric uncertainty Lack of control group
Agopyan et al., 2019 [24]	*N* =136 female nutrition and dietetic students Only those whose scores indicated diagnosis of orthorexia or ED were included	18 to 30 *M* = 20.97	N	Convenience	ORTO-11 Eating Attitude Test (EAT-40) Body composition analysis	A total of 70.65% of participants met criteria for orthorexia Mean ORTO-11 = 27 Significant negative correlation between EAT-40 and ORTO-11 No significant difference found in body composition and orthorexia prevalence	Cross-sectional design ORTO-11 psychometric uncertainty Convenience sampling Lack of control group
Aktürk et al., 2019 [25]	*N* = 558 female nursing participants	Not stated	N	Convenience	ORTO-15	A total of 73.5% of participants met criteria for orthorexia Mean ORTO-15 = not specified Orthorexic symptoms were significantly related to: age, gender, class, perceived health, dieting status, and weight satisfaction Diet behaviours and socio-demographic factors were significantly associated with orthorexia All factors except dieting significantly predicted orthorexia Weight satisfaction had the greatest impact on orthorexia prevalence followed by age and gender	Cross-sectional design ORTO-15 psychometric uncertainty Convenience sampling Lack of control group
Asil and Sürücüoğlu, 2015 [26]	*N* = 117 dietitians	Range not stated *M* = 34	N	Stratified	EAT-40 ORTO-15 Maudsley Obsessive Compulsive Inventory (MOCI) BMI calculated	Participants with ORTO-15 scores < 40 had significantly higher EAT-40 and MOCI scores Mean ORTO-15 = 39.7 There was a significant negative correlation between ORTO-15 scores and EAT-40 scores and BMI A total of 41.9% of participants received ORTO-15 scores congruent with orthorexia cut-off point Participants with a BMI less than 25 had significantly lower EAT-40 scores but higher ORTO-15 scores	Small sample size Lack of control group ORTO-15 psychometric uncertainty
Bağci Bosi et al., 2007 [27]	*N* = 318 resident doctors	20 to >30 *M* = 27.2	N	Convenience	ORTO-15 BMI calculations	A total of 45.5% received scores suggestive of orthorexia Mean ORTO-15 = 39.8 There was no significant sex difference in ORTO-15 scores Food selection in 20.1% males and 38.9% females said to be influenced by nutrition/health in mass-media Females reported significantly greater care for their physical appearance and weight control which they reported controlling through their food selection A total of 20.1% of male participants and 38.9% of females reported that health programmes affected their food selection Participants who reported trying to control their weight had significantly greater number of orthorexic behaviours than those who were not	Psychometric uncertainty of ORTO-15 Lack of control group Convenience sampling
Busatta et al., 2021 [28]	*N* = 30 dietetic students, 30 participants with a diagnosed ED and 30 control students	18 to 29 *M* for Dietetics = 22.70 *M* for ED = 21.89 *M* for controls = 22.41	Y	Convenience	ORTO-15 Eating Disorder Examination Questionnaire Symptom Checklist-90-Revised	ED participants had significantly greater orthorexic tendencies than other groups Mean ORTO-15 for ED participants = 33.23 Dietitian and controls had no significant difference in ORTO-15 scores Orthorexia positively correlated to ED psychopathology in ED groups but not controls	The presence of psychiatric disorders beyond EDs were only excluded in the ED sample. Causing a significant confounding variable and sample heterogeneity Psychometric uncertainty of ORTO-15 Convenience sampling
Caferoglu and Toklu, 2022 [29]	*N* = 1429 dietitians and dietetic students	18 to 65 *M* = 23.20	N	Stratified	ORTO-11 EAT-26	Prevalence of orthorexia was 59.8% Mean ORTO-11 = 26.31 Prevalence was significantly greater in dietic students than dietitians Eating disorders were reported to cause a fivefold risk of orthorexia Graduated dietitians had a 33.1% lower risk of orthorexia compared to students Having an obese BMI significantly decreased prevalence of orthorexia Significant negative correlations were found between EAT-26 and ORTO-11 scores across sample	Cross-sectional design ORTO-11 psychometric uncertainty Only 9.2% of participants were male, low male generalisability Anthropometric data were self-reported, decreasing reliability Lack of control group
Depa et al., 2016 [30]	*N* = 188 nutrition science and 268 economics students	18 to 27 *M* = 21.7	Y	Convenience	Düsseldorf Orthorexia Scale	A 3.3% prevalence of orthorexia, an additional 9% were at risk of orthorexia No significant difference between age, semester of study, or sex Female nutrition students scored significantly higher on the subscales ‘‘avoidance of additives’’ and ‘‘supply of minerals’’	Unequal group sizes Cross-sectional design Small sample size The two population samples were obtained two years apart, increasing the possibility of potential heterogenous sociocultural confounding factors
Dittfeld et al., 2016 [31]	*N* = 430 dietetic and physiotherapy students	18 to 26 Dietetic *M* = 21.5 Physiotherapy *M* = 20.9	N	Convenience	Bratman Orthorexia Test (BOT) Author made questionnaire to assess attitude toward food, nutrition, and self-perception	A total of 26.6% of dietetics students and 14.9% of physiotherapy students were classified as ‘health food fanatics’. There was a significant group difference Significance relationship between orthorexia and BMI for dietetic students. Higher BOT scores were associated with increased BMI 88.2% of dietetic and 52% of physiotherapy students declared that they noticed changes in their attitude towards food after becoming students	Cross-sectional design Convenience sampling Author made questionnaire with no reported psychometrics Lack of control group
Erol and Özer, 2019 [32]	*N* = 298 medical students	Not stated	N	Convenience	ORTO-15 EAT-40	A total of 76.2% had orthorexic tendencies Mean ORTO-15 = 36.1 A total of 11.1% had eating disorders Obese students had less orthorexic tendencies Individuals who received information from nutritionists has significantly greater prevalence of eating disorders	Lack of information on method provided in research article Lack of control group Convenience sampling
Farchakh et al., 2019 [33]	*N* = 627 medical students Recruited from seven universities	Range not stated *M* = 21.81	N	Stratified	ORTO-15 EAT-26 Hamilton Anxiety Rating Scale A	Higher EAT-26 scores significantly associated with lower ORTO-15 scores Mean ORTO-15 = not specified Higher anxiety scores significantly associated with higher ORTO-15 scores No significant difference between ORTO-15 scores and age, BMI, gender, or university	Weak effect sizes ORTO-15 psychometric uncertainty Lack of control group
Fidan et al., 2010 [34]	*N* = 878 medical students	16 to 29 *M* = 21.3	N	Convenience	ORTO-11 EAT-40 BMI calculations	Males had significantly higher orthorexic tendencies Younger participants had significantly greater orthorexic tendencies Age, sex, height, and EAT-40 scores, significantly affected the ORTO-11 scores There was a significant negative relationship between BMI and ORTO-11 scores A 43.6% prevalence of orthorexia Mean ORTO-11 = 27	ORTO-11 psychometric uncertainty Convenience sampling Cross-sectional design Lack of control group
Freire et al., 2020 [35]	*N* = 60 physical practitioners Sampled from gym locations	Range not stated *M* = 26.58	N	Stratified	EAT-26 ORTO-15 Body Shape Questionnaire Scale of Dedication to Exercise	A total of 80% of practitioners met ORTO-15 cut-off for orthorexia Mean ORTO-15 = not specified A total of 80% participants demonstrated a low degree of exercise addiction Women were significantly more dissatisfied with their bodies than men Younger practitioners had significantly higher indication of EDs Individuals dissatisfied with their body demonstrated significantly higher presence of EDs and higher addiction to exercise No significant correlation between body dissatisfaction and orthorexia Significant negative correlation between orthorexic behaviour and presence of ED	Small sample size, limited power ORTO-15 psychometric uncertainty Lack of control group
Grammatikopoulou et al., 2018 [8]	*N* = 176 undergraduate nutrition and dietetics students	18 to 30 *M* = 21.7	N	Convenience	Food Diary Eating and Appraisal Due to Emotion and Stress questionnaire Modified Yale Food Addiction scale BOT	A total of 4.5% of participants met criteria for food addiction; 68.2% met criteria for orthorexia Nil gender differences Those who met criteria for orthorexia demonstrated increased BMI but reduced energy and saturated fat intake Linear regression demonstrated orthorexic behaviour was associated with significant increases in BMI, waist size and energy intake	Cross-sectional design Small sample size, minimising power Convenience sampling Lack of control group
Guglielmetti et al., 2022 [36]	*N* = 169 health-science students, 192 economic-humanistic students, 218 sport science students, and 92 dietetics and nutrition students	Range not stated *M* = 21	Y	Convenience	ORTO-15	A total of 31.2% orthorexia prevalence with a cut-off of <35 Mean ORTO-15 = not specified No difference according to BMI or sex No difference in prevalence according to area of study Dieting was a significant risk factor for the development of orthorexia in health-scientific, economic-humanistic and sport science students	Unequal groups Did not assess for the presence of other EDs Only first year students were surveyed, decreasing generalisability ORTO-15 psychometric uncertainty Possible concerns for validity due to self-reported data
Karakus et al., 2017 [37]	*N* = 208 nutrition and dietetics students	Not stated	N	Convenience	ORTO-11	Orthorexic tendencies significantly greater in males Orthorexia significantly greater in students living with their families Orthorexia did not differ according to: smoking, alcohol, chronic disease, BMI, diet, or nutritional supplements Mean ORTO-11 = 16.5	Cross-sectional design Convenience sampling ORTO-11 psychometric uncertainty Use of singular assessment tool Lack of control group
Kinzl et al., 2006 [38]	*N* = 283 female dieticians	22 to 66 *M* = 36.3	N	Stratified	BOT Three-Factor Eating Questionnaire—German Version	A 12.8% orthorexia prevalence A total of 34.9% of participants demonstrated “sone orthorexic behaviours” A total of 8.8% of participants reported increased self-esteem from eating healthy food; 4.6% felt guilt when straying from their diet Participants who met cut-off for orthorexia indicated significantly greater restraint and higher disinhibition of eating than those who did not meet cut-off criteria	Small sample size Lack of control group Unable to generalise results for males
Korinth et al., 2010 [10]	*N* = 123 first year nutrition students, 96 higher semester nutrition students and 114 non- nutrition first year university students	Range not stated First year students *M* = 22.5 Higher semester students *M* = 25.7	Y	Stratified	Dietary restraint scale and the disinhibition scale from the Eating Behaviour Questionnaire ORTO-10 Short Food Frequency Questionnaire	No significant differences between orthorexia and gender, BMI or age Mean ORTO-10 = not specified Nutrition students showed significantly higher dietary restrain than controls Dietary restraint significantly higher in first year students compared to later semester students Nutrition students had significantly greater rigid and flexible control over food than controls No significant difference in orthorexic behaviours between nutrition students and the control group in the first two semesters of study Tendency of orthorexic behaviours significantly decreased in the nutrition students between the first/second semester and the seventh semester of study The food frequency score did not differ between nutrition students and the control group in the first two semesters. Food selection significantly improved in the nutrition students towards the end of their programme	Cross-sectional approach suggests support for changes in eating behaviour throughout duration of study however due to the design causal conclusions cannot be drawn ORTO-10 psychometric uncertainty
Malmborg et al., 2017 [39]	*N* = 118 exercise science students and 89 business students	19 to 29 *M* = 22.8	Y	Convenience	Short Form-36 Health Survey International Physical Activity Questionnaire ORTO-15	Exercise students reported significantly more body pain than business students but no significant difference in their exercise engagement A total of 76.6% of all students met cut-off for orthorexia Mean ORTO-15 = 36.7 Significantly higher orthorexia in exercise students	Cross-sectional design ORTO-15 psychometric uncertainty Convenience sampling
Plichta and Jezewska-Zychowicz, 2019 [40]	*N* = 547 health major university students & 573 university students in other majors Sample drawn from seven universities	18 to 35 *M* = 21.4	Y	Stratified	ORTO-15 Health Concern Scale Food Frequency Questionnaire	Health-related degrees had significantly lower ORTO-15 scores, indicative of greater orthorexic behaviours Mean ORTO-15 = 36.6 Presence of orthorexia symptoms did not differ by gender or BMI	Cross-sectional design ORTO-15 psychometric uncertainty
Plichta et al., 2019 [41]	*N* = 547 health majors students and 573 non-health major students Sampled from seven universities	18 to 35 *M* not stated	Y	Stratified	ORTO-15 The Questionnaire of Body Particular Parts and Parameters Satisfaction Food Frequency Questionnaire	When utilising a cut-off of 40, 75% of participants met criteria for orthorexia. At a cut-off of 35, 28.3% met orthorexic criteria Mean ORTO-15 = 36.6 Health students were significantly more likely to have orthorexia then students in non-health majors Women were significantly more dissatisfied with their bodies than men; however, there was no significant association between orthorexia and sex Students with orthorexia were significantly more satisfied with their upper body than non orthorexic students No significant group differences were identified with lower body satisfaction Nil identified relationship between orthorexia and BMI	Bias of self-reporting BMI Study was conducted during lecturers, limiting anonymity and jeopardising validity of responses
Sanlier et al., 2016 [42]	*N* = 900 university students Students were from social sciences, physical and mathematical sciences, and health-related degrees	17 to 23 *M* = 20.37	Y	Convenience	EAT-40 ORTO-15	No significant differences in EAT-40 scores according to gender or BMI EAT-40 scores were significantly higher in social science students There was no difference in ORTO-15 scores according to degree Mean ORTO-15 = 39.06 Females had significantly greater prevalence of orthorexia compared to males Individuals with subclinical and pathological eating attitudes measured by EAT-40 had increased orthorexic behaviours	ORTO-15 psychometric uncertainty Cross-sectional design Convenience sampling
Selçuk and Çevik, 2020 [43]	*N* = 568 nursing students	17 to >22 *M* = 21.29	N	Convenience	ORTO-11	Significant negative correlation between ORTO-11 scores and dietary supplement use Mena ORTO-11 = 37.94 Greater orthorexic behaviours were associated with supplement use Vitamin B12, iron, and vitamin C were the most used supplements	Significant gender imbalance with 72% of participants being female Data on BMI, physical activity, and supplement use were self-reported, confounding reliability of results Convenience sampling Lack of control group Cross-sectional design ORTO-15 psychometric uncertainty
Tremelling et al., 2017 [44]	*N* = 636 dietitians	Not stated	N	Stratified	ORTO-15 Eating disorder examination questionnaire (EDE-Q)	A total of 49.5% of participants were at high risk of orthorexia Mean ORTO-15 = 39.3 A total of 12.9% at high risk of ED Participants with a current or previous ED and those with scores in the range of orthorexia scored significantly higher on the EDE-Q and had lower BMIs than other participants Of those who previously or currently had an ED diagnosis, 59.6% met criteria for orthorexia based on ORTO-15	The eating disorder group included individuals at any stage of recovery, substantial group heterogeneity ORTO-15 psychometric uncertainty Lack of control group
Villa et al., 2022 [45]	*N* = 90 nutrition and dietetic students	Range not stated *M*—22.2	N	Convenience	ORTO-11 Spanish Version Instagram use was measured from phone data collection Attitudinal, physical and social demographics via author-made questionnaire	A 23.3% prevalence of orthorexia Mean ORTO-11 = 26.8 Several risk variables for developing orthorexia were identified: being in the second year of study, coming from a charter school, living with only one other person, or living alone Significant negative correlation between BMI and Orthorexia for women Both sedentary and high levels of physical activity was significantly associated with orthorexia risk Those in the lowest and highest Instagram use groups demonstrated significantly greater risk of orthorexia	Small sample size Convenience sampling ORTO-11 psychometric uncertainty Nil psychometric properties were reported for author-made questionnaire
Yılmazel, 2021 [46]	*N* = 969 candi-date doctors and nursing students	Range not stated *M* = 21.4	N	Conven-ience	ORTO-15 Social media addiction scales	A total of 78.8% of participants were addicted to social media; 62.2% had orthorexic tendencies Mean ORTO-15 = not specified Significantly greater orthorexic tendencies amongst the high/very high social media addiction group Significantly greater orthorexia prevalence in doctors than nurses No significant differ-ence in orthorexia based upon chronic problems, health per-ception, physical activ-ity, weight satisfaction, or BMI Significantly great prevalence of or-thorexia in those who did not have a diet programme	ORTO-15 psycho-metric uncertainty Lack of control group Convenience sam-pling

**Table 2:** Study merit.

Author and Date	Presence of Control Group	Matched Control Group	Representative Sample	Is Orthorexia Well Defined	Do the Assessments Have Sound Psychometrics	Are Limitations Appropriate	Total Score
Abdullah et al. (2020) [23]			*	*		*	3
Agopyan et al. (2019) [24]				*	*	*	3
Aktürk et al. (2019) [25]				*			1
Asil and Sürücüoğlu (2015) [26]			*		*		2
Bağci Bosi et al. (2007) [27]				*			1
Busatta et al. (2021) [28]	*	*		*	*	*	5
Caferoglu and Toklu (2021) [29]			*	*	*	*	4
Depa et al. (2016) [30]	*			*	*	*	4
Dittfeld et al. (2016) [31]				*	*		2
Erol and Özer (2019) [32]					*		1
Farchakh et al. (2019) [33]			*	*		*	3
Fidan et al. (2010) [34]				*	*		2
Freire et al. (2020) [35]			*		*		2
Grammatikopoulou et al. (2018) [8]					*	*	2
Guglielmetti et al. (2022) [36]	*			*		*	3
Karakus et al. (2017) [37]				*			1
Kinzl et al. (2006) [38]					*		1
Korinth et al. (2010) [10]	*	*	*		*	*	5
Malmborg et al. (2017) [39]	*	*		*		*	4
Plichta and Jezewska-Zychowicz (2019) [40]	*		*	*		*	4
Plichta et al. (2019) [41]	*		*	*	*	*	5
Sanlier et al. (2016) [42]	*			*	*	*	4
Selçuk and Çevik (2020) [43]				*			1
Tremelling et al. (2017) [44]			*	*	*	*	4
Villa et al. (2022) [45]						*	1
Yilmazel (2021) [46]						*	1

The asterisks (*) denote that the specific criterion or element (e.g., “Matched Control Group,” “Representative Sample," etc.) was met or present in that particular study.

## Data Availability

Not applicable.
